# Impact of remote experimentation, interactivity and platform effectiveness on laboratory learning outcomes

**DOI:** 10.1186/s41239-021-00272-z

**Published:** 2021-07-08

**Authors:** Krishnashree Achuthan, Dhananjay Raghavan, Balakrishnan Shankar, Saneesh P. Francis, Vysakh Kani Kolil

**Affiliations:** 1 Center for Cybersecurity Systems and Networks, Amrita Vishwa Vidyapeetham, Amritapuri, Kollam, 690525 India; 2 Department of Mechanical Engineering, Amrita School of Engineering, Amrita Vishwa Vidyapeetham, Amritapuri, Kollam, 690525 India

**Keywords:** Virtual labs, Remote trigger, Interactivity, Mechanics of solids, UTM, Learning outcome, Transactional distance theory

## Abstract

Access and personalized instruction required for laboratory education can be highly compromised due to regulatory constraints in times such as COVID-19 pandemic or resource shortages at other times. This directly impacts the student engagement and immersion that are necessary for conceptual and procedural understanding for scientific experimentation. While online and remote laboratories have potential to address the aforementioned challenges, theoretical perspectives of laboratory learning outcomes are critical to enhance their impact and are sparsely examined in the literature. Using Transactional Distance Theory (TDT), this paper addresses the gap through a case study on Universal Testing Machine (UTM). By comparing physical (PL-UTM) and remotely triggerable (RT-UTM) laboratory platforms, the structure and interactions as per TDT are analysed. Characterization of interactivity between remote learners and instructors disclose indicative parameters that affect transactional distances and aid in conceptual understanding in remote laboratory learning environment. An extensive pedagogical study through development of two instruments towards assessing conceptual understanding and perception of platform effectiveness that was conducted both on physical laboratory and RT-UTM showed: (1) remote users conducted experiments 3 times more frequently (2) completed assignments in 30% less time and (3) had over 200% improvement in scores when RT-UTM platform was integrated into mainstream learning.

**Supplementary Information:**

The online version contains supplementary material available at 10.1186/s41239-021-00272-z.

## Introduction

Laboratories are indispensable in pre-university and university level science and engineering education (De Jong et al., 2013; Brinson, 2015; Feisel & Rosa, 2005; Ma & Nickerson, 2006). Hands-on learning and trouble-shooting skills acquired in laboratory experimentation complement the classroom lectures (Satterthwait, 2010; Heradio et al., 2016; Tzafestas et al., 2006; Clough, 2002; Gillet et al., 2003). Accreditation agencies such as ABET (Accreditation Board for Engineering and Technology) define their accreditation criteria for programs as those enabling graduates with an ability to apply knowledge of mathematics, science and engineering, ability to design a system, conduct experiments, analyze and interpret data, an ability to function on multi-disciplinary teams, an ability to identify, formulate, and solve engineering problems and so on (ABET, 2018; Anwar & Richards, 2018). Similar requirements from regulatory bodies such as All India Council for Technical Education (AICTE) and National Board of Accreditation (NBA) include curricula that enhance engineering knowledge, problem analysis, design and development of investigative approaches to complex problems, modern tool usage and so on (AICTE, 2019). Such curricula are expected to meet the ever-growing industry requirements from a knowledge, skills, and attitudes perspective (Seifan et al., 2020; Gleich et al., 2020). Physical laboratories are fundamental in equipping students of science and engineering with the requisite skills for real-world problem solving.

One of the challenges in physical laboratories is that when student numbers are large and they are grouped together to perform experiments, student chances of success at meeting expected learning outcomes can be compromised due to lack of individualized learning. These are further exacerbated in times such as COVID-19 pandemic (WHO, 2020) as well as at other times where resource shortages exist (Cooper & Ferreira, 2009; Stamovlasis et al., 2006).

Mechanical engineering education is a field that relies heavily on laboratory education as a foundational pillar in providing the practical skills required for engineering graduates. Specifically, the mechanics of solids concepts are best studied in a laboratory setting where boundary conditions and applied forces can be closely scrutinized to study compatibility with theoretical assumptions (Jara et al., 2008). One of the main experimental hardware in the mechanics of solids laboratory is the Universal Testing Machine (UTM) (Mao et al., 2019) . This was chosen for detailed study as part of this work : (1) UTM experiments being part of the mandatory model curriculum set by regulatory bodies (AICTE, 2019) and (2) the UTM hardware is the crux of mechanics of solids laboratory for undergraduate civil, mechanical and aerospace engineering courses. UTM is a load application and extension measurement machine that can be used for a wide variety of material testing applications such as tension, compression, double shear and bending. A critical drawback of the UTM hardware and strain gauge instrumentation is that they are prone to mishaps such as overloading, test initiation at wrong strain rates, and gauges peeling off due to specimen mishandling (Lowe et al., 2009). As a result, the strain gauges and related UTM hardware are shielded from student access and allowed to be computer-controlled only by the instructor in order to run pre-programmed test schedules for the student groups to observe, thus dampening the learning outcomes.

In such instances remote laboratories (Cooper & Ferreira, 2009) not only allow greater accessibility, but have the potential to bridge the gaps in development of laboratory skills by allowing individual students to work with the physical laboratory (PL) equipment remotely (Achuthan et al., 2020; Lowe et al., 2013). The widespread reach of information technology has widened the scope of laboratory education (Achuthan et al., 2018; Kolil et al., 2020; Achuthan et al., 2017; Raman et al., 2011). There are considerable benefits of remote laboratories, namely, enabling greater flexibility, lower costs and greater resource sharing. The environment in which learning takes place, whether online or face to face, involves a complex array of factors that influence learner satisfaction and achievement (Aguilera-Hermida, 2020; Bali & Liu, 2018).

While physical laboratories have been well researched from theoretical perspective through multiple types of student interactions (Komorek & Kattmann, 2008; Wei et al., 2018), such studies on remote laboratories are sparse (Wei et al., 2019). The significant contributions of this paper are (1) designing a remote lab such as RT-UTM that allows greater flexibility in performance of 5 experiments in comparison to its equivalent physical UTM lab, (2) capturing the structure and interactivity of PL-UTM and RT-UTM through the Transactional distance theory (TDT) and ultimately (3) conceptual understanding in remote vs physical and integrated remote and physical lab environments through (4) the development and implementation of two survey instruments.

### Theoretical framework

**Fig. 1:**
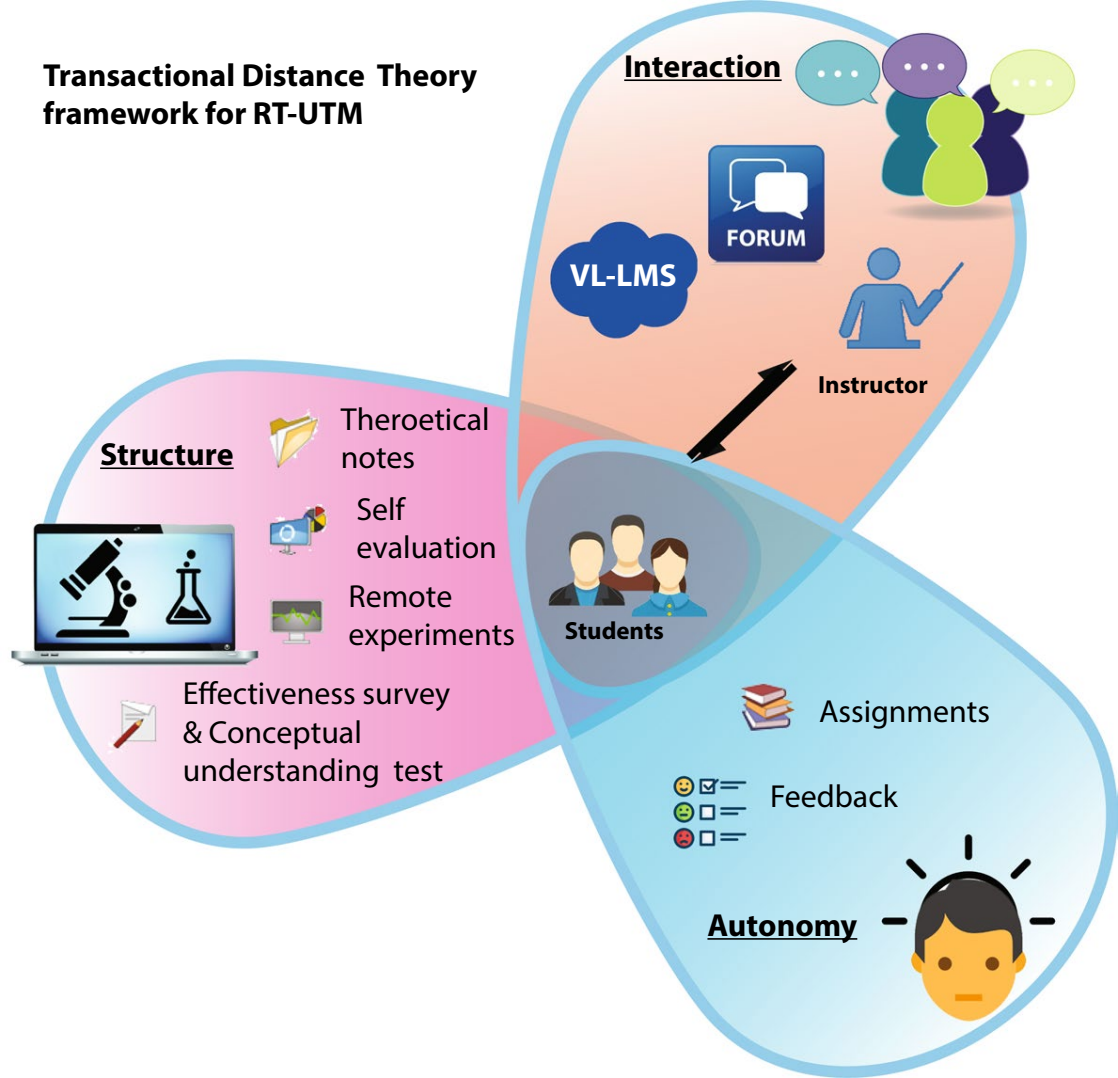
Transactional distance theory framework for RT-UTM

Moore’s Transactional Distance Theory (TDT) has proven to be an immensely useful theoretical framework to study the effectiveness of remote laboratories in engineering education (Moore, 1973, 1991; Garrison, 2000; Jung, 2001; Murphy and Collins 1997; Goel et al., 2012; Delgaty, 2018). The distance between the instructor and the student is substantial in remote education (Tirado-Morueta et al., 2018) and TDT explains and quantifies the learning relationship between instructor and student in the remote learning situation (Delgaty, 2018). The high transactional distance between teacher and students may contribute to feelings of isolation and reduced motivation and engagement in students (Moore, 1991). According to Moore, the three constructs of TDT are (1) Structure, (2) Interaction (or Dialogue), and (3) Learner Autonomy (Moore and Kearsley 2011). Structure represents the rigidity or flexibility of the instructional materials and methods, interaction represents the interaction between the instructor, students and machine (or equipment) (Moore, 1989; Lowe et al., 2008; Sher, 2009), and autonomy represents the nature and degree of self-directedness of the students. Identifying the level of structure required, facilitating interaction and encouraging individual learner autonomy is demanding as the greater the structure and the lower the interaction, the more autonomy the student must demonstrate. The interactions that are part of the learning process (Goel et al., 2012) help to improve the conceptual understanding of the students (Wei et al., 2019). Successful transactional distance environments depend on the teacher providing opportunities for interaction and ‘appropriately’ (Moore, 1993) structured learning materials. The greater, and faster, and more involved the level of interaction, the lower the level of psychological feeling of separation there would be (Wei et al., 2019; Moore & William, 2007). Efficiently structured content that utilizes latest technologies and increased interaction promote effective online learning (Sun & Chen, 2016). Literature suggests that transactional distance is directly proportional to structure and inversely proportional to interaction (Saba, 2012; Demir Kaymak & Horzum, 2013). Higher learner autonomy implies less structure required to reduce the transactional distance (Kearsley & Moore, 2012). Compared to traditional learning, online learning happens through viewing pre-recorded videos via the internet. Online platforms are highly flexible (low structure) and allow interactive learning environments (Jung, 2000; Jung, IS 2000; Pauls, 2003). The key objectives of this work included assessing and comparing the PL-UTM and RT-UTM features with respect to the transactional distance, which is inversely proportional to the effectiveness of the remote laboratory (Lindsay et al., 2007). The TDT framework for RT-UTM depicted in Fig. 1 included multiple structural components such as detailed theoretical descriptions, self-evaluation questions, remote user-interface for defining experimental parameters, live result observation windows with calculator to monitor output parameter evolution, and instant data-set export function. The in-built virtual lab learning management system (VL-LMS) and discussion forum aids student-instructor (S-I) and student-student (S-S) interactions. Finally, the conceptual understanding analysis gauges the flexibility of the laboratory platform’s structure in aiding student learning.

## Methods

### Design of physical and remote UTM experimentation

**Fig. 2:**
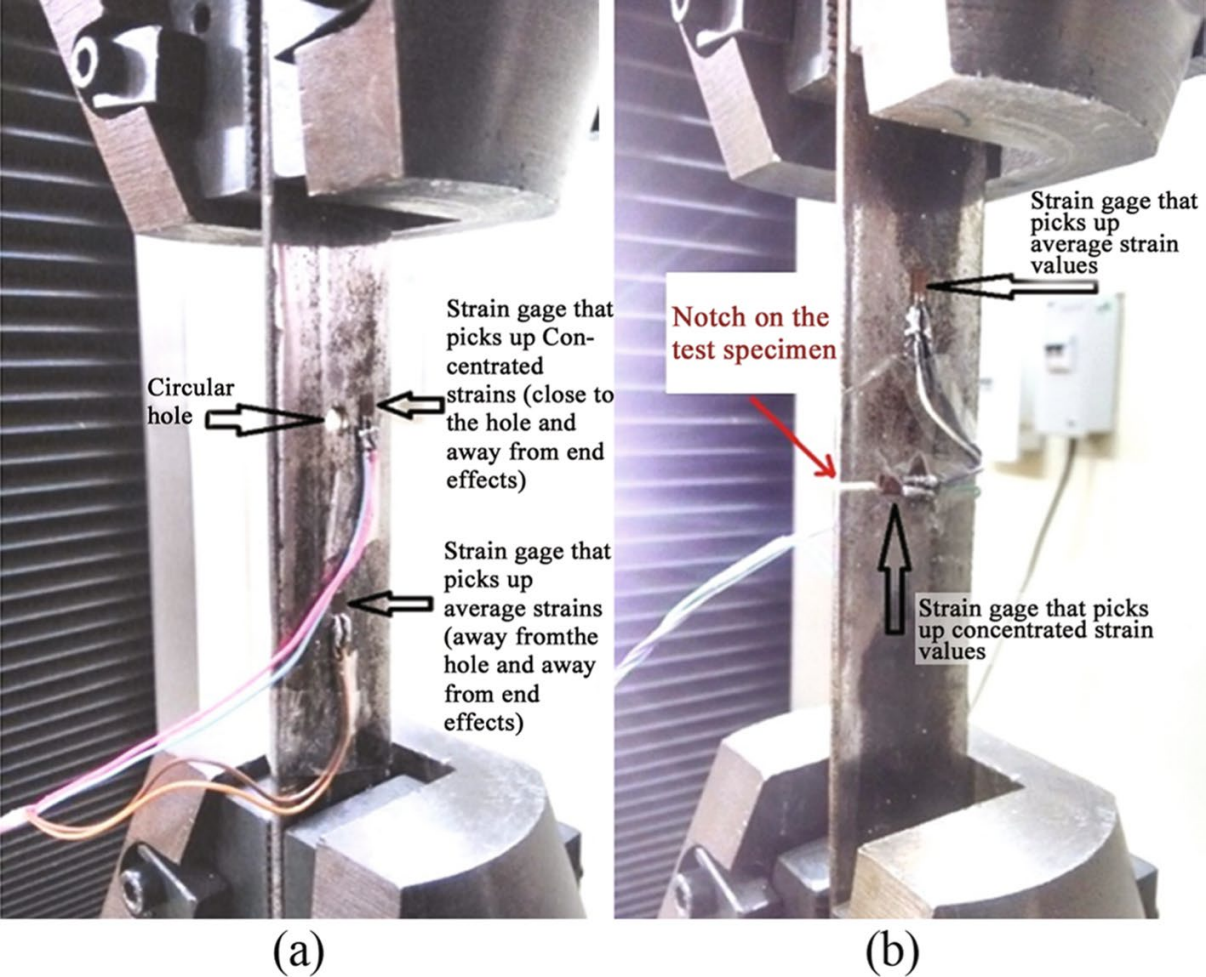
Specimen loaded in the UTM for experimentation.** a** Specimen with circular hole and** b** specimen with notch

The focus of this study was experimentation with UTM, which is used for load application and deformation measurement involving tension, shear, bending, and compression. The bouquet of experiments within UTM included determination of Young’s modulus, determination of Poisson’s ratio, study of stress concentrations on a plate with a hole and notch, and study of Saint Venant’s principle. A sample specimen setup in the UTM is shown in Fig. 2.

The UTM that is predominantly used in the mechanics of solids undergraduate laboratory course is utilized by batches of students to perform experiments physically (hence termed PL-UTM). Typically students spend between 2–3 h in the physical lab. An hour is spent on instruction of theory and procedure followed by 30 min to perform the initial measurements of length, diameter of specimen, and strain gauge wiring. This is followed by physical experimentation for about an hour. Most of the doubts regarding theory and procedure are raised and interactively addressed during the experimentation when students see the actual test and come to terms with what is happening. This takes time away from focusing on the actual test data during the live experiment. They perform the analysis asynchronously after laboratory hours. Thus physical laboratory courses taught in STEM (Science, Technology, Engineering, and Mathematics) education are rigid in their structures due to various constraints such as limited time to complete experiments, safety protocols that are required to be followed and in some cases insufficient infrastructure or resources for personalized learning, which lead to inadequate hardware interaction (student-machine, S-M) which limits the learning effectiveness (Lal et al., 2019; Lowe et al., 2008).

**Fig. 3:**
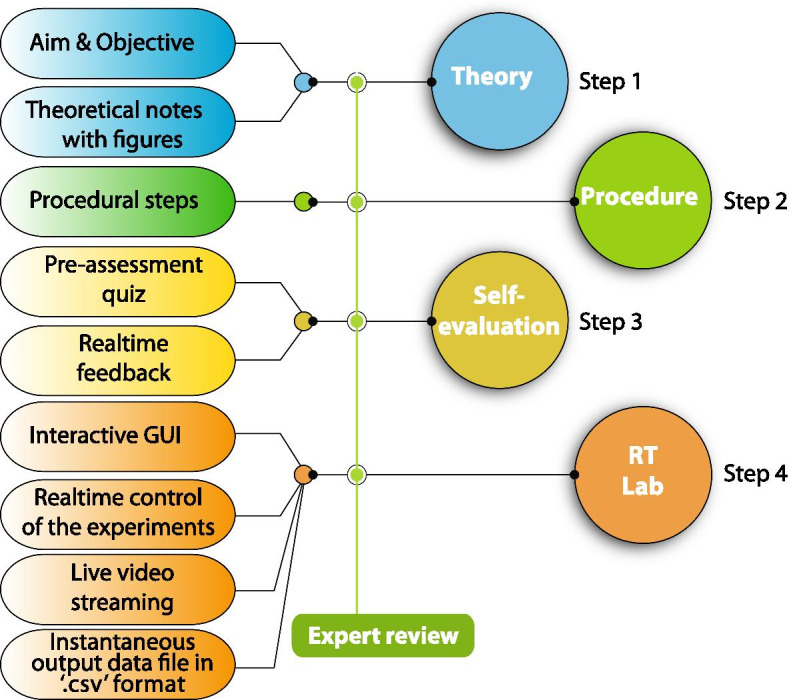
Design of RT-UTM platform

The design of educational activities in the Remotely triggerable UTM (i.e., RT-UTM) are shown in Fig. 3. The features, flexibility and procedural elements of the platform were prepared to help learners exercise increased autonomy. Concepts are introduced highlighting the underlying theory prior to the start of virtual experimentation. A set of pre-assessment questions were provided in the RT-UTM to emphasize and gauge understanding of key concepts. Through an interactive graphical user interface (GUI) learners are not only able to, in real time, control the experiment but also view the live streaming of the experimentation. Additionally they also have the facility to download and analyze the data. Learners are able to repeat experiments, imbibe the educational content and analyze data in a far more synchronous fashion compared to PL-UTM. The details of the remote experimentation set up and user interface of the experiment are described in the following sections.

**Fig. 4:**
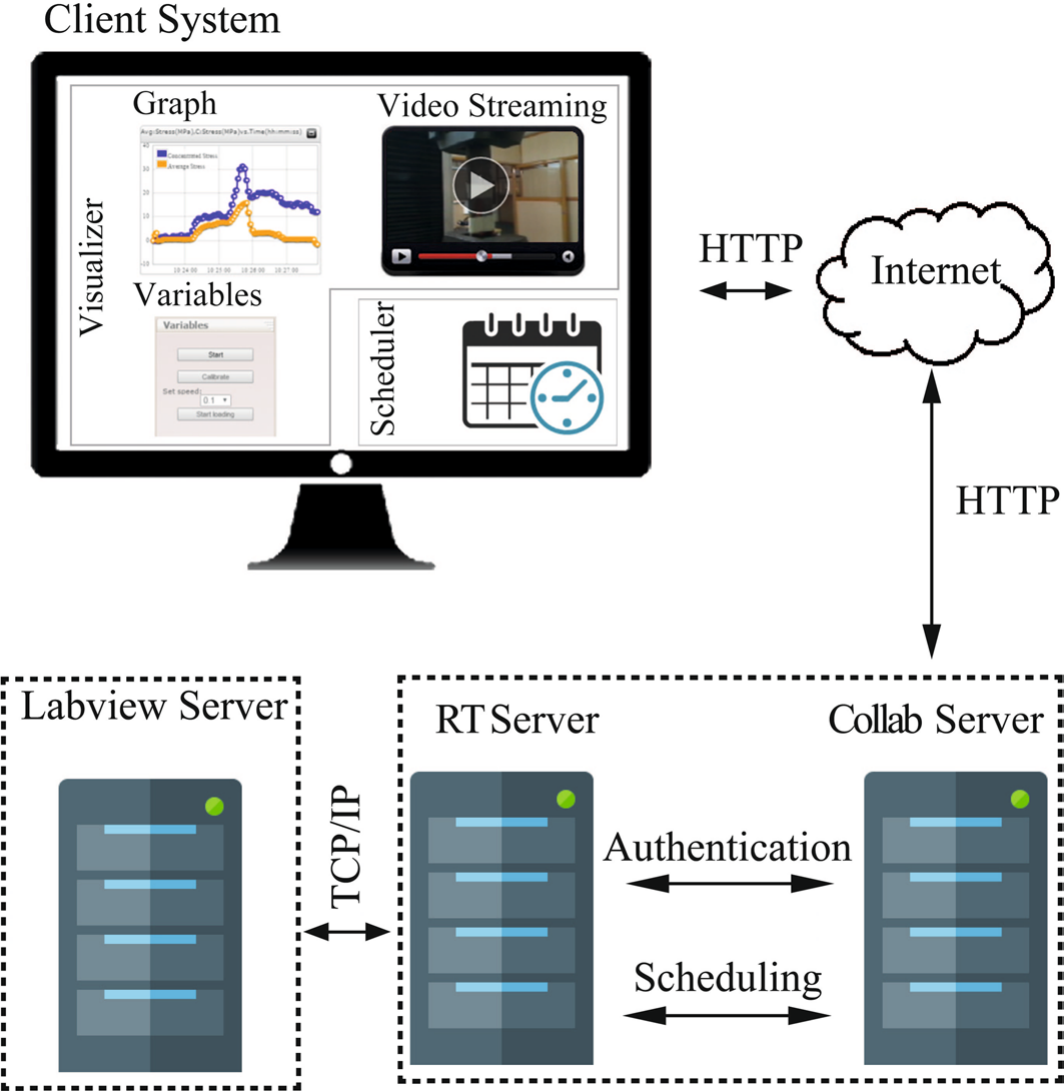
Architecture of remote triggered experimental setup

#### Architecture of remote experimental setup

Schematic representation of client user architecture of the developed remote experimental setup is represented in Fig. 4. The components of the system include: (1) Client system—where the users can access the RT experiments via internet (2) Collab server—which is used to host the RT-UTM website and authenticate the registered users based on user id and password (3) Remote Trigger (RT) Server—which hosts the scheduler for accessing RT-UTM experiment to accommodate multiple users. If users access RT-UTM simultaneously, the scheduler automatically informs the requester and schedules them for another time. Once the scheduler confirms a time or date, the experiment is made available for that particular registered user. (4) Labview Server—which provides necessary connections with the RT-UTM apparatus and collects the measurements. When the user triggers the RT-UTM experiment, Labview server controls the UTM machine with respect to the parameters set by the user and acquires the readings from the instrumentation integrated with RT-UTM.

**Fig. 5:**
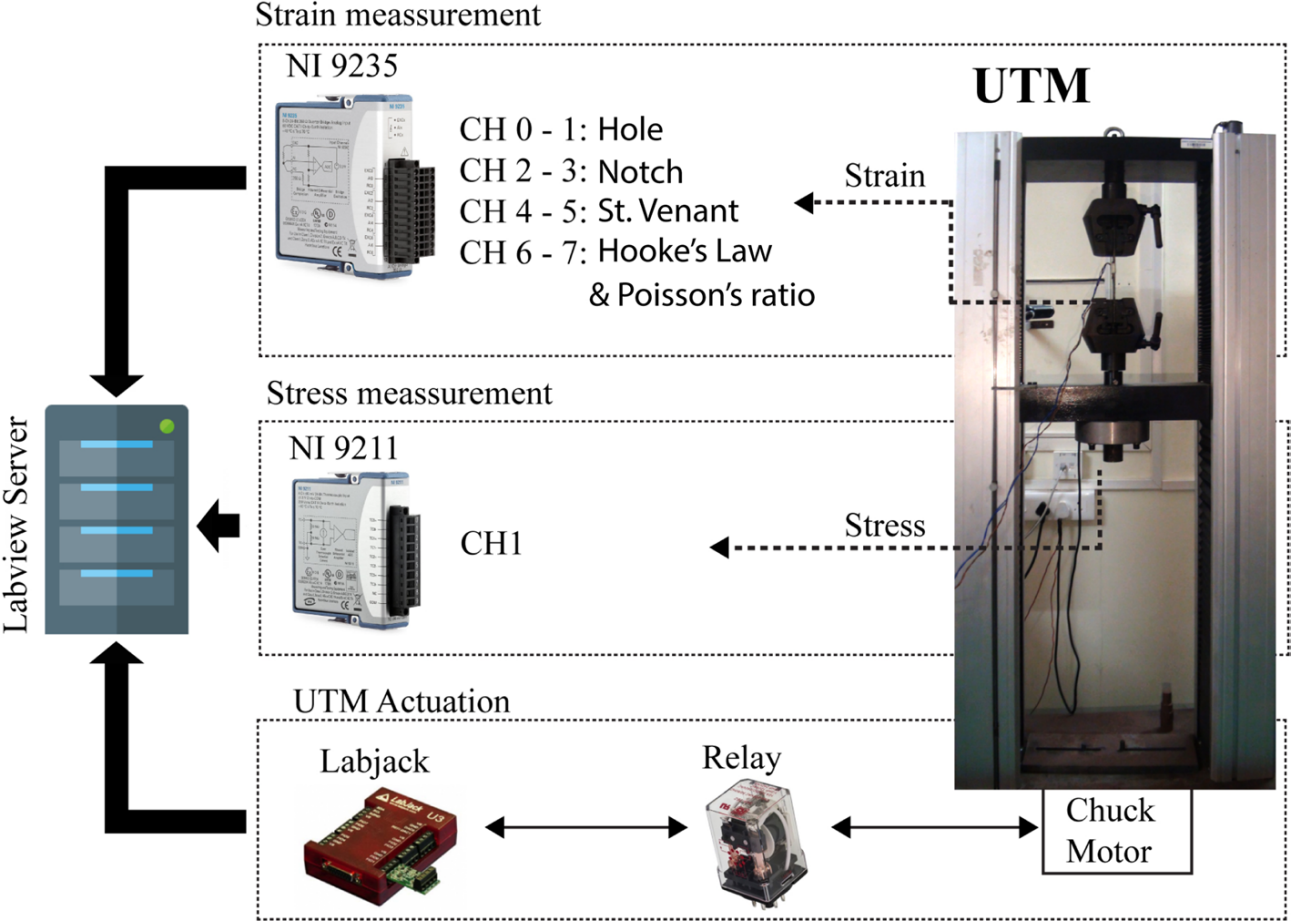
Schematic representation of instrumentation hardware

The process flow of request and access to RT-UTM takes place as follows: When the user accesses the RT-UTM setup via internet, a HTTP request is sent to the Collab server. The collab server authenticates the users and connects them to the RT server. The RT server does the scheduling and permits the connection with Labview server. The Labview Server allows access of the hardware interface to the users (Raman et al., 2011; Nedungadi et al., 2011),

Data Acquisition and Control Hardware (DAQ), placed between UTM and Labview server, controls and executes the experiment as per the user’s request. Figure 5 represents the block diagram of the data acquisition and control hardware. DAQ consists of two measurement hardware [NI 9235 (NI-9235, 2018) and NI 9211 (NI-9211, 2018)] developed by National Instruments Corporation and a Labjack for controlling the UTM load actuator (chuck motor) which is used to apply load to the specimen. NI 9235 is used to measure the strain from strain gauges attached to the specimen in a quarter-bridge configuration. NI 9211 is used to measure the force from the load cell for stress calculation.

#### Remotely triggerable-UTM user interface

**Fig. 6:**
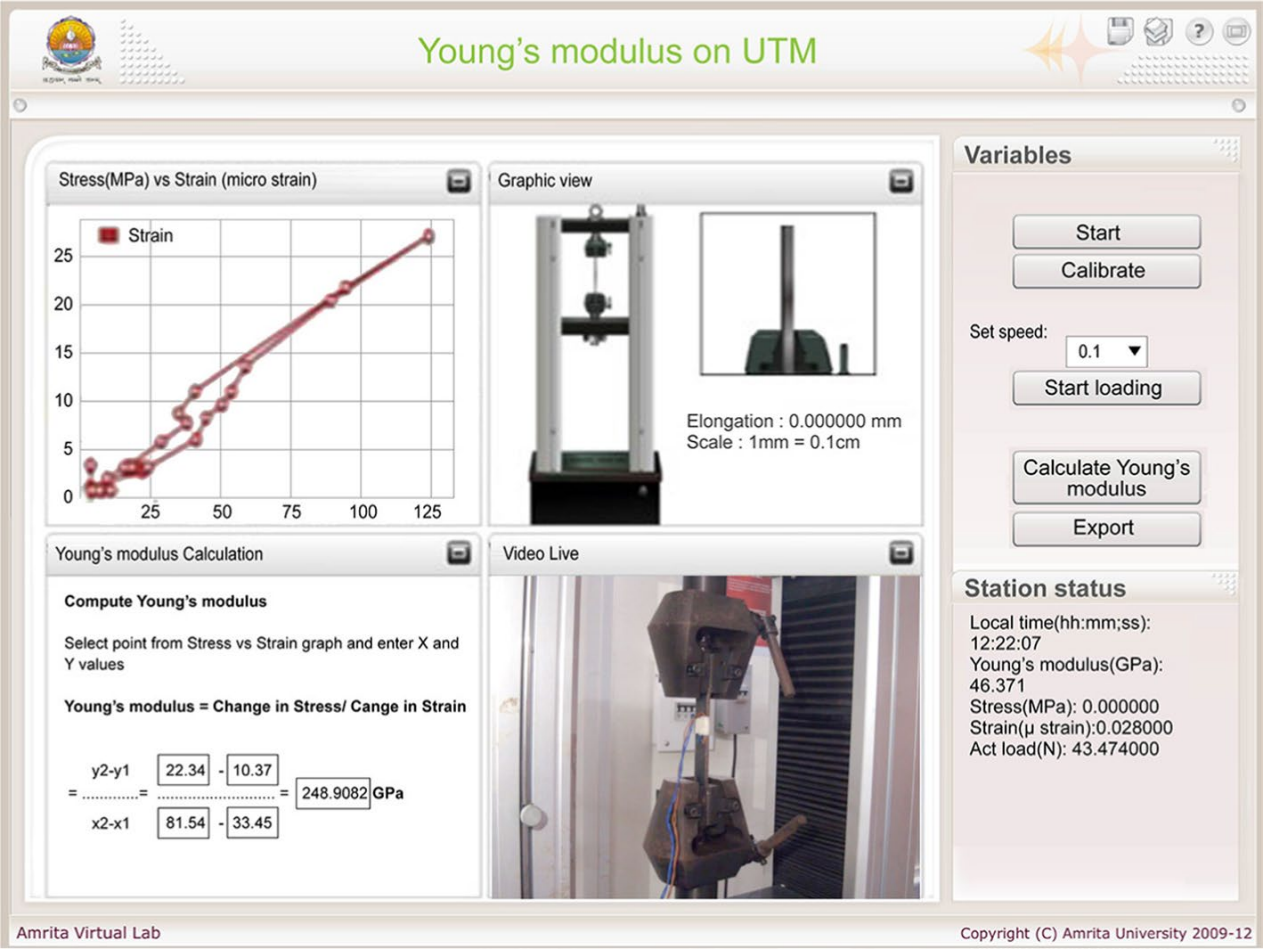
Client-side UI for Young’s modulus experiment

The typical graphical User Interface (UI) built for these experiments is shown in Figure 6. It has four sub-windows portraying (1) the live plot of stress (MPa) versus strain (microstrain), (2) a graphical animation view of the specimen elongating, (3) a calculator window that helps compute parameters of interest from the experiment and (4) real time video of the actual remote experiment.

The live plot allows the user to select the points on the stress-strain graph and the calculator window allows to identify the slope of the curve between these points as a real-time, interactive means to check the results while the experiment is running live. The ‘.csv’ (Comma-separated values) data export option at the end of the experiment provides the final data set used for post-processing the results. In addition to the animated view, real-time view provides a realistic feel of the experiment even while performing it remotely. The station status shows the current test value being obtained. The experiment is safe-guarded by allowing user inputs that are within the safe limit of both the specimen (below yield stress) and UTM (50 kN capacity).

On the right side of the UI there are two blocks; Variables - to perform the experiment, and Station status - to monitor the time, stress, strain, actual load etc. Variables that control the RT-UTM has five buttons labeled ‘Start’, ‘Calibrate’, ‘Start loading’, ‘Calculate’ and ‘Export’. The ’Export’ option is used to download the final data set of the experiment run in ‘.csv’ format for post-processing and completion of experiment objectives. In addition, the Variables block allows the user to set the speed of the UTM load on the specimen (0.1–0.3 mm/min) in order to study load rate effects on elastic properties, which motivates the experimental endeavor and differentiates the remote lab from computer simulation.

To perform this experiment, the user needs to click the ‘Start’ button and establish the connection between the RT-UTM and the client system. The ‘Calibrate’ button will be activated as soon as the connection is established. Calibration of strain gauges and initialization of the load cell to start the loading process is then initiated. Post calibration, the user can select the speed (0.1–0.3 mm/min) to start loading the specimen. The RT-UTM restricts the continuous load applied to 2kN to avoid specimen yielding and permanent deformation. Upon reaching this limit, the system automatically unloads the specimen. The calculate button calculates the experimental value of the quantity being investigated (for example: experimental value of Young’s modulus for the Young’s modulus experiment) from the experimental readings. The Export button allows the user to export the data into a ‘.csv’ file format for further analysis.

### Performing RT-UTM experimentation

The learning platform is comprehensive in that it not only allows remote experimentation, but also provides detailed preparatory material to the users that helps them with (1) understanding the theory and concepts behind the experiments (2) a detailed step-by-step experimental procedure, (3) video tutorials of how the experiments are performed in physical laboratory, (4) self-evaluation quizzes to evaluate themselves on conceptual knowledge prior to start of the experiment, (5) assignment questions on computing parameters through RT-UTM. Immediate feedback from the self-evaluation helps users to correct their understanding before performing the experimentation. The assignment questions cover the complete aspects of UTM experiment and were prepared by mechanical engineering faculty members who have not only taught UTM concepts but are also subject matter experts. These assignments enhance student learning by facilitating application of knowledge gained through RT-UTM Laboratory to real world problems. Since Remote triggered experiments are restricted to one user at a time, a scheduler is integrated and that helps to accommodate multiple requests sequentially on the RT-UTM.

On the host side, the specimens are changed manually once a day according to the schedule of the experiment. The scheduler in the procedure tab helps to arrange one experiment every day to minimize human intervention. No further human presence is allowed in the remote lab at the time of experimentation and the performance of the hardware is also monitored remotely. The experiments performed are described as follows.

**Fig. 7:**
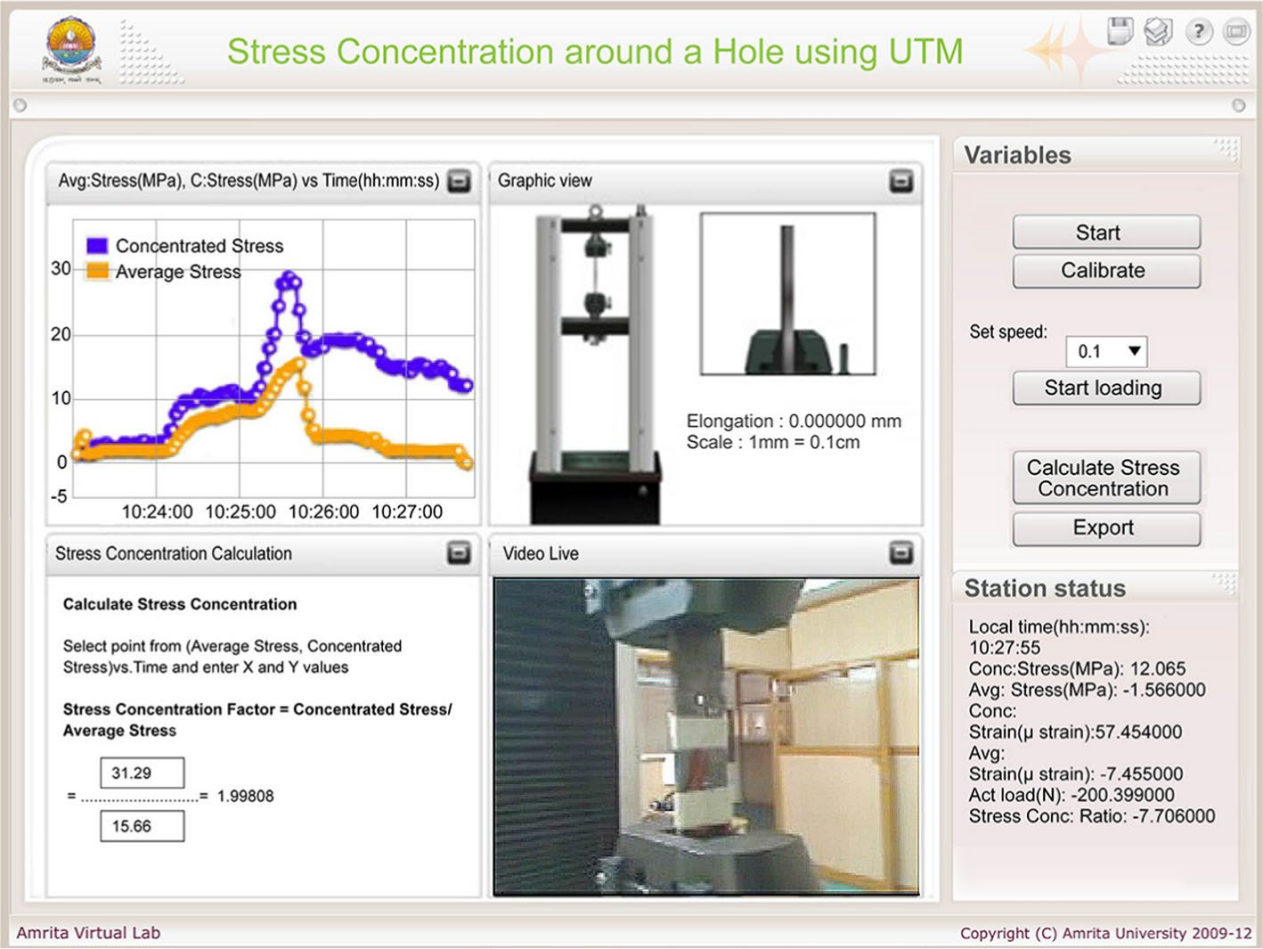
Client-side UI to study stress concentrations on a plate with a hole

#### Determination of Young’s modulus

In this experiment students study the relationship between stress and strain. Young’s modulus is calculated from the slope of the stress-strain plot in a UTM experiment. In the physical laboratory, groups of students perform the experiments with the help of an instructor. In some cases, the instructor performs the experiment and groups of students merely observe the experimentation, limiting student participation to data collection only. The RT-UTM serves as a collaborative learning tool that improves student participation and engagement thereby complementing the physical lab by providing one-on-one hardware access. The UI of RT-UTM’s Young’s modulus experiment is shown in Fig. 6. All experiments are designed for the study of linear elastic mechanics of solids.

#### Determination of Poisson’s ratio

**Fig. 8:**
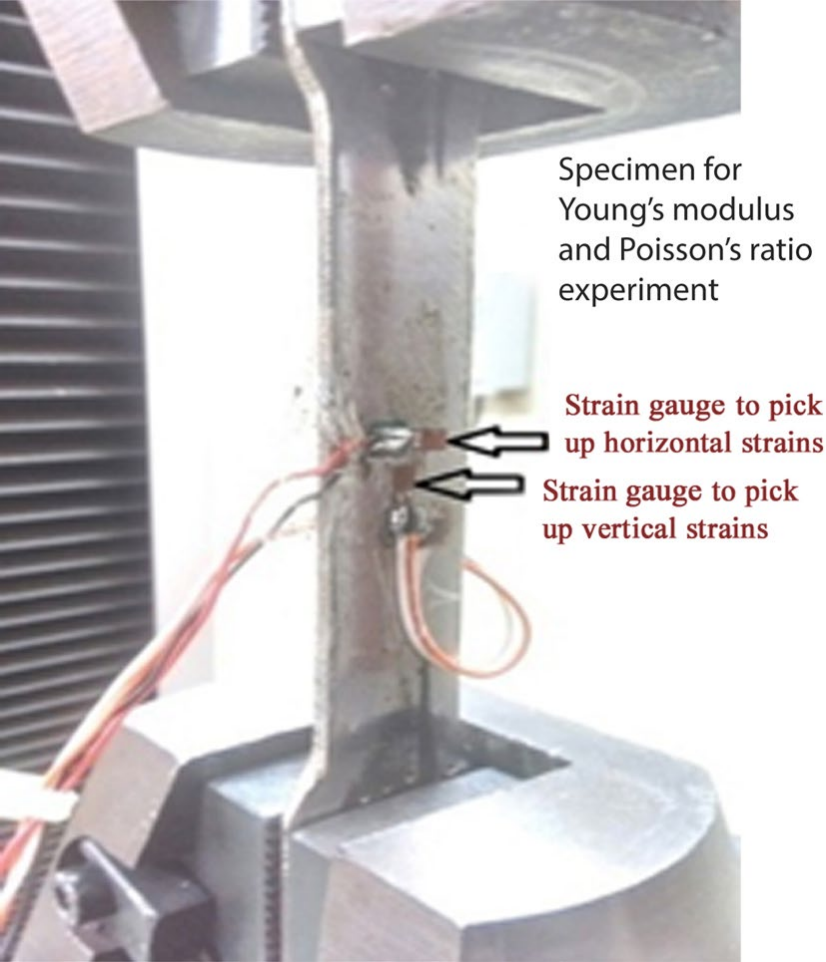
Specimen with strain gauges loaded in the UTM for Poisson’s Ratio Experiment

Poisson’s ratio is determined by the ratio of lateral strain to the longitudinal strain. It is the measure of lateral compression or expansion happening perpendicular to the direction of specimen loading relative to the longitudinal deformation. Client side graphical UI for the experiment is similar to Fig. 7. Figure 8 represents the specimen loaded in the UTM machine to determine Poisson’s ratio. There are two strain gauges attached to the specimen for measuring the lateral and longitudinal strains, as per the recommended ASTM E132-17 experimental setup for rectangular cross-section specimens (ASTM 2017). Readings are displayed in the client side UI graph. Users can calculate the value of Poisson’s ratio using the graph select tool.

#### Study of stress concentrations on a plate with a hole

The objective of this experiment is to measure the average and concentrated stress on a mild steel (0.05–0.25% carbon) specimen under tension due to the effect of a circular hole in the specimen. There are two strain gauges used for this experiment. The first strain gauge is placed adjacent to the hole to pick up the stress concentration and the second gauge is placed away from the hole to pick up the average or nominal stress. The graphical UI of this experiment is shown in Fig. 7.

**Fig. 9:**
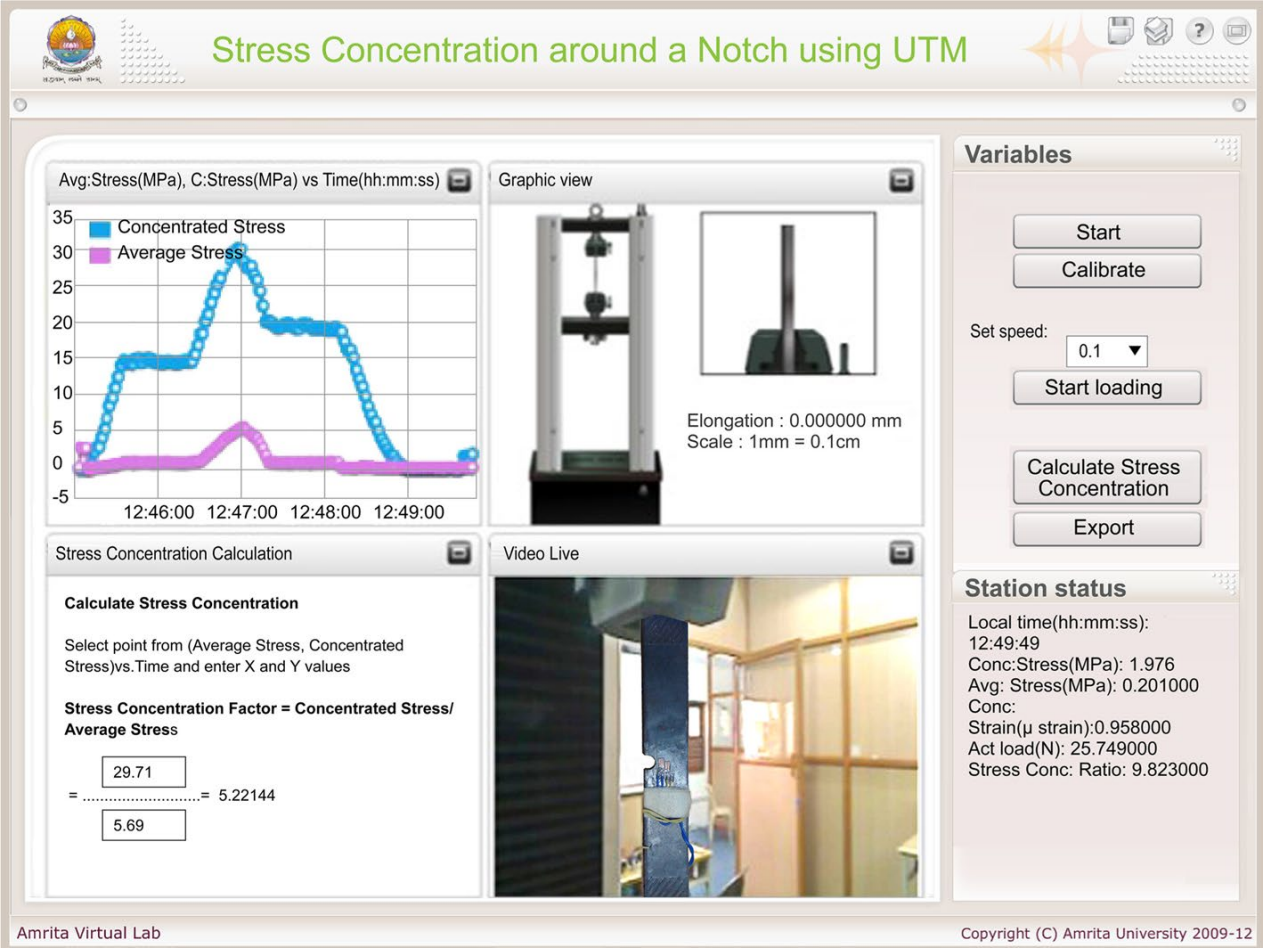
Client-side UI to study stress concentrations on a plate with a notch

#### Study of stress concentrations on a plate with a notch

The graphical UI of the experiment is shown in Fig. 9. UI and Experimental procedure is same as described in the previous section ‘study of stress concentrations on a plate with a hole’. The user can calculate the stress concentration factor by entering readings in the input field and by pressing the ‘Calculate Stress concentration’ button.

#### Study of St. Venant’s principle

**Fig. 10:**
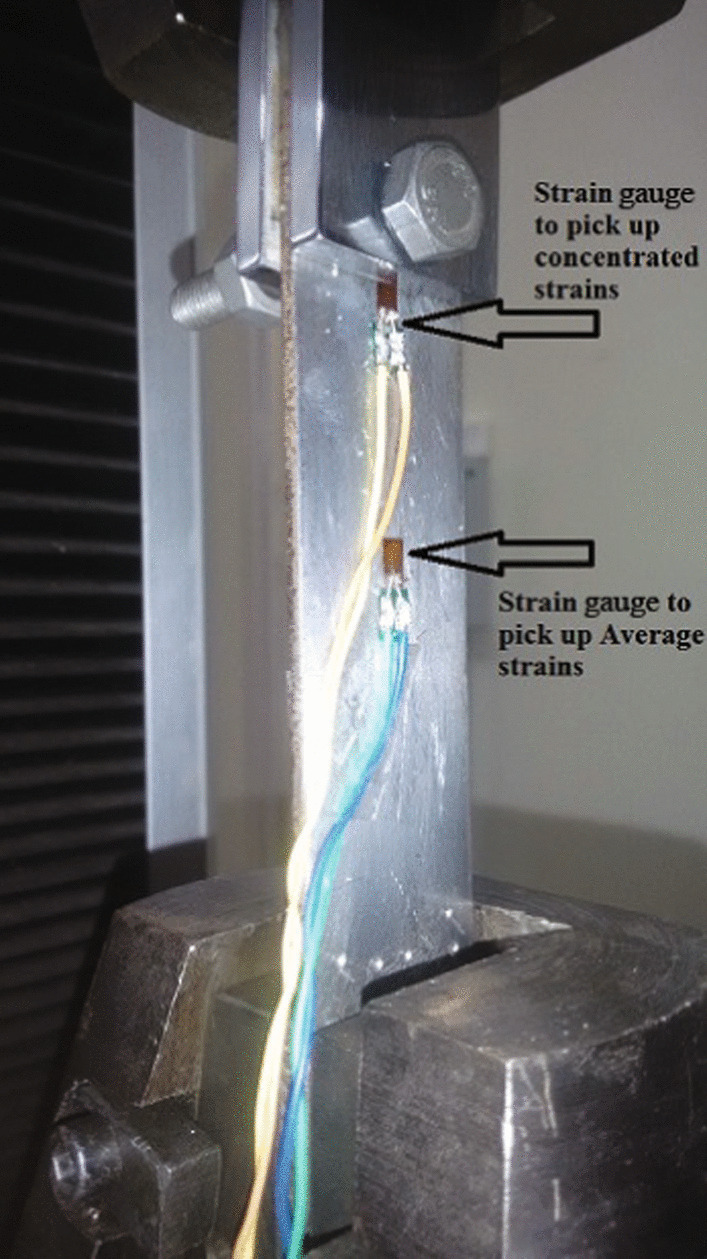
Strain gauge locations on specimen for St. Venant’s Principle

In the St.Venant’s Principle experiment, two strain gauges are fixed. One near the central portion of the specimen to pick up the peak stress in the uniform stress distribution region and the another near the upper grip of the RT-UTM to pick up the peak stress in the non-uniform stress distribution region near the load application point, as shown in Fig. 10. A special fixture was designed to apply the concentrated load which is not possible with the UTM grips as they distribute load over the grip surface. The fixture allows for a bolt to go through a hole in the specimen, and the bolt is held in place with a nut across two guide plates which do not contact the specimen but act as spacers. The two guide plates converge into the UTM grip (Fig. 10), allowing the load to be applied via the bolt shaft onto the specimen in a concentrated manner. The UTM is then switched on and the specimen is subjected to tensile load. The respective strain values obtained from both the gauges are measured and then plotted with respect to time.

As per St. Venant’s principle, the peak stress across the specimen width will be concentrated near the point of application of the load, although the average stress along the uniform cross section far enough away from the load application point remains constant. The further the distance from the point of application of load, the more uniform the stress is distributed across the cross section.

### Structure of the learning platform

The benefits of the RT-UTM design in terms of structure can be seen in Table 1. The rigidity or flexibility of the RT-UTM structure can be assessed in terms of variety, individualization, formality, media use, cognitive load, visualization, functionality and usability (Huang et al., 2015). Variety is enhanced in RT-UTM by multiple content formats (audio, video, animation) and modes of interaction (S-I, S-S, S-M). Individualization is also improved in RT-UTM by the self-paced nature of the platform which allows multiple experiment repetitions under different load rates and two-way communication (learning management system), while maintaining the same structural formality in terms of course outlines and objectives. The ease of media use (interface and experiment controls) provided by RT-UTM enriches the learning experience by promoting better S-M interaction (Lowe et al., 2008; Lal et al., 2019, 2018). The cognitive load on RT-UTM is expected to be lower with the use of ICT and absence of physical hazards. Step-by-step procedure is provided for the instrumentation and controls which, if not followed sequentially, will prevent activation of the controls for the next stage of the experiment, and guide the user to revisit the procedural steps until the correct operational sequence is performed. This feature is typically absent in physical labs, allowing erroneous test initiation and controls, which lead to incorrect measurements, safety concerns, and higher cognitive load requirements. Visualization is characterized by a spatially well-designed RT-UTM interface while ensuring the same functionality as a physical lab through the interaction afforded by the learning management system (LMS). Usability is improved in RT-UTM through easy navigation of the online platform.

**Table 1:** Structure in remote triggered-UTM and physical lab-UTM

No	Parameter	PL-UTM	RT-UTM
1	Variety	Low	High
2	Individualization	Low	High
3	Formality	Same	Same
4	Media use	Low	High
5	Cognitive load	High	Low
6	Visualization	Low	High
7	Functionality	Same	Same
8	Usability	Low	High

**Fig. 11:**
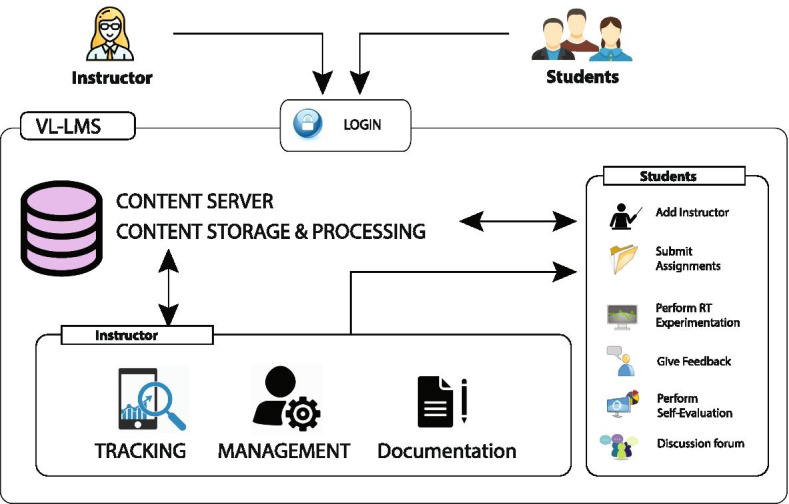
Block diagram of VL-LMS

**Fig. 12:**
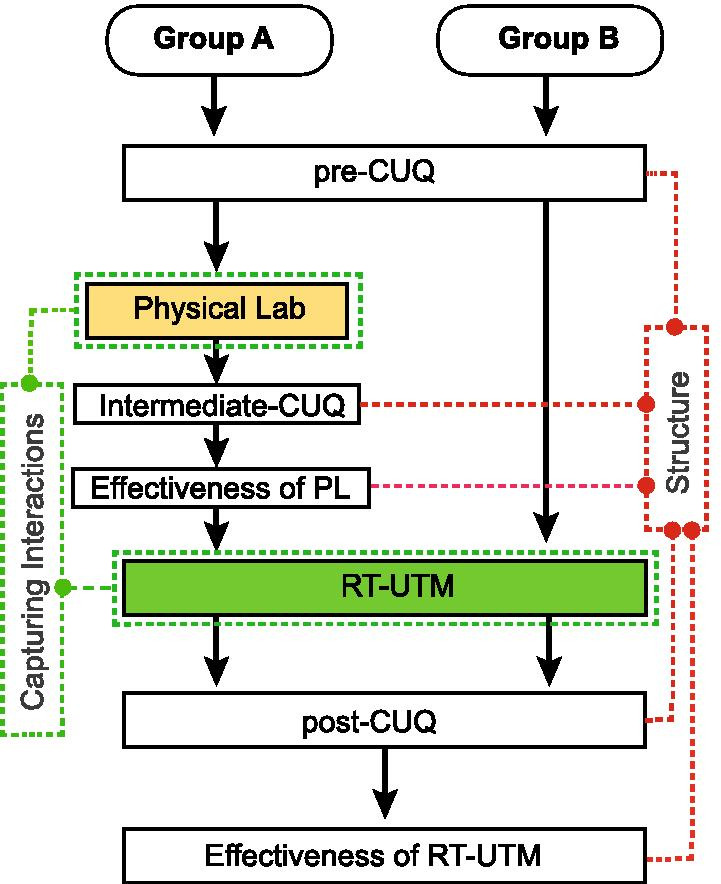
Methodology for assessing RT experimentation effect

### Virtual lab learning management system (VL-LMS)

Enhanced interaction between students and teachers is aided by the integration of LMS within the Virtual Lab framework (Nedungadi et al., 2011). The primary users of VL-LMS are either the students or the instructors with each having specific access privileges. Figure 11 shows the schematic representation of the VL-LMS with user privileges. Instructors can prepare and manage the assessment questions, and assign them to groups of students, schedule the assessment time and track individual student responses. On the other hand, student privileges include: entering into group or individualized chats with peers or instructors, viewing and submitting assignments, performing RT experimentation, self evaluation tests, and providing feedback. VL-LMS stores student interactivity data in the server and this includes their responses to questions, the number of attempts taken to answer questions, total time spent online and so on. The student-student (S-S) and student-instructor (S-I) interactions were captured while performing the experiments in physical and RT-UTM laboratories as shown in (Fig. 12). In RT-UTM, interactions were captured using VL-LMS whereas, in the physical lab the interactions were captured from the observations of students and feedback from multiple instructors.

### Assessment of learning in PL-UTM and RL-UTM platform

For the assessment of the PL-UTM and RT-UTM in meeting the learning outcomes, an experimental study (Fig. 12) was conducted on two groups of students, group A (GA, N = 50) and group B (GB, N = 50), from the second year undergraduate (UG) mechanical engineering course. The participants were grouped randomly to avoid any bias and both groups had undergone an UG level theory course on Mechanics of Solids. Two types of assessments were conducted for this study. One was conceptual understanding (CU) of governing principles behind experiments through a conceptual understanding questionnaire (CUQ) and the second assessment was the effectiveness of use of the learning platform through a survey instrument. Ethics approval was gained for the data obtained from the Institutional review board.

#### Conceptual understanding questionnaire (CUQ)

A questionnaire was designed to evaluate the conceptual understanding gained from both environments i.e., the physical laboratory (PL) and RT-UTM. The learning objectives of the UTM laboratory experiments were to (1) identify the relation between stress and strain, (2) analyse the stress concentration factor and (3) measure the modulus of elasticity of the material. The CUQ consisted of 14 multiple choice questions (MCQ) (Additional file 1) developed with the help of instructors with more than a decade of teaching experience in mechanics of solids. These questions were designed to test students’ understanding of Young’s modulus, stress concentration due to the presence of hole and notch on the specimen, and Poisson’s ratio. Each correct answer earned 1 point and a student could score a maximum of 14 from the CUQ questionnaire.

As shown in Fig. 12 a pre-CUQ assessment was conducted for GA and GB students to assess their conceptual understanding before proceeding to laboratory experimentation. After the pre-CUQ assessment, GA students were directed to perform the UTM experiments in PL while GB students were sent to a computer laboratory that had the RT-UTM interface to perform RT-virtual experimentation. In the PL, students usually perform the experiments in groups whereas in RT-UTM, they perform the experiments individually within the online RT platform. After completion of PL experiments, the GA group was assessed using the CUQ evaluation, referred to as Inter-CUQ. After this intermediate evaluation, the GA group was also asked to perform the RT-UTM experiments from the computer laboratory. Seven days were allotted to both groups to complete all the UTM experiments detailed in the methodology section. A post-lab assessment called post-CUQ was administered to both groups (GA and GB) after completion of the RT-UTM experiments.

#### Effectiveness of RT virtual laboratory platform

The formulation of this survey questionnaire was inspired by a similar work on control laboratories (Vargas et al., 2011) and remote laboratories (Nickerson et al., 2007; May et al., 2016). An instrument with 15 questions (Additional file 2) was formulated (5 point Likert scale from strongly disagree = 1 to strongly agree = 5) to evaluate the following criteria, namely comprehension without supervision, well-defined procedure, user interactivity, user convenience and impact on learning. These criteria enable assessment of the structure and interaction of the RT platform in accordance with transactional distance theory. The reliability of the instrument was characterized using the Cronbach’s Alpha method (Gliem and Gliem 2003), and the instrument was found to be reliable ($\alpha$ $=$ 0.79). Immediate feedback (effectiveness of learning platform) was requested from both groups separately in the form of a survey after performing the PL-UTM and RT-UTM experimentation using the LMS (Nedungadi et al., 2011; Rapuano and Zoino 2006) where a supervisor monitored the time taken for completing the experimentation and feedback.

## Results and discussion

There are distinct differences between physical and RT laboratory experimental platforms. The differences include (1) experiment availability and flexibility for repetitions, (2) experimental time, (3) type of interactivity between students, instructors and equipment. Usually PL experimentation takes two to three hours to complete due to the need for the instructor to explain the experimental setup and procedure prior to manually loading the specimen into the machine and applying the load. These requirements are minimized in RT-UTM platform, due to the specimen being pre-loaded into the RT-UTM machine. In PL, most often, experiments are performed in groups because of limited availability of UTM machines and laboratory time. This diminishes opportunities for individualized learning and adequate laboratory exposure for students. In contrast, in RT-UTM, experiments are performed individually and can be repeated until conceptual clarity is obtained. The increased individualization and student-machine interaction provided by one-on-one experimentation with the equipment, and the added flexibility of experiment repetition without time constraints, help to lower the transactional distance. The RT-UTM is able to easily demonstrate its impact on learning by addressing practical testing issues, namely, its ability to see stress concentration factors in real-time due to high values of stress near a discontinuity and observe real-time disturbances in the linear elastic stress-strain curve due to the slip between the specimen and the grip.

### Student interactivity

VL-LMS platform allows students to have interactions with instructors (S-I) as well as peers (S-S). A comparison of such interactivity in RT-UTM and Physical-UTM laboratory sessions are tabulated in Table 2.

**Table 2:** Interactions observed in RT-UTM and Physical Lab-UTM

	RT-UTM	Physical lab
Number of students participated	50	50
Average Time taken to complete assignment (Minutes)	37.32 ± 9.95	60 ± 15.49
Average number of attempts taken (Maximum 5 attempts in RT-UTM)	2.98 ± 1.43	1
Number of Students participated in S-S discussion	17 (34%)	30 (60%)
Number of Students participated in S-I discussion	30 (60%)	10 (20%)
Time spent on student-machine interaction (Minutes)	25	5
Number of questions asked by students	37	22
Questions related to theory	19	8
Questions related to experiment and its procedural steps	18	14

The data shows remote students worked and re-worked on experiments three times more than those in physical labs (PLs). There was also significant difference in the time taken for completion of assignments between the the two modes with remote students taking 30% lesser time. The S-S interactions were more in the PLs and this corroborates with earlier findings that physical proximity promotes interaction (Corter et al., 2011; Lindsay et al., 2007; Messman and Jones-Corley 2001; Park et al., 2017; Fila and Loui 2014). On the other hand, the S-I interactions increased with RT-UTM (Lal et al., 2019). On analysis of the interactions and questions, it was found that students who were learning remotely had more questions overall on the theory, experimentation and procedural processes. This is indicative of the impact of an immersive environment that allows students to experiment more frequently, think independently and seek answers quickly. These results corroborate with a prior study on usage of LMS to capture student-instructor interactions in remote laboratories (Tirado et. al 2018). In addition, S-M interaction also increased in RT-UTM due to one-on-one experimentation on the hardware. Thus, RT-UTM has enabled more S-I and S-M interactions in comparison to PL-UTM, leading to lower TD with RT-UTM.

**Table 3:** t-test result of pre-CUQ, intermediate-CUQ and post-CUQ assessment of Group A and Group B

Group	Mean	SD	df	GA-inter *t/p*	GA-post *t/p*	GB-pre *t/p*	GB-post *t/p*
GA-pre	3.80	1.85	49	$-$ 16.34/ 0.00*			
					$-$ 21.99/0.00*		
						0.87/0.39	
							$-$ 19.00/0.00*
GA-inter	8.86	1.21	49		$-$ 9.22/ 0.00*		
						19.27/0.00*	
							$-$ 6.06/0.00*
GA-post	11.54	1.86	49			23.70/ 0.00*	
							0.91/0.37
GB-pre	3.50	1.66	49				$-$ 20.29/ 0.00*
GB-post	11.18	2.21	49				

*significant at p <0.05

### Conceptual understanding (CU)

Assessment of learning through design and development of conceptual understanding questionnaire (CUQ) helped quantify understanding of governing scientific phenomena behind the experiments. A pre- and post-CUQ assessment was conducted before and after the laboratory experimentation (Fig. 12). An independent sample t-test is used to compare the results of Group A (GA) and Group B (GB). The pre-test results in the Table 3 shows that there is no significant difference (*p* = 0.39) in the score of GA-pre (M = 3.80, SD = 1.85) with GB-pre (M = 3.50, SD = 1.66), implying student groups GA and GB were similar in their CU prior to the experimentation.

**Fig. 13:**
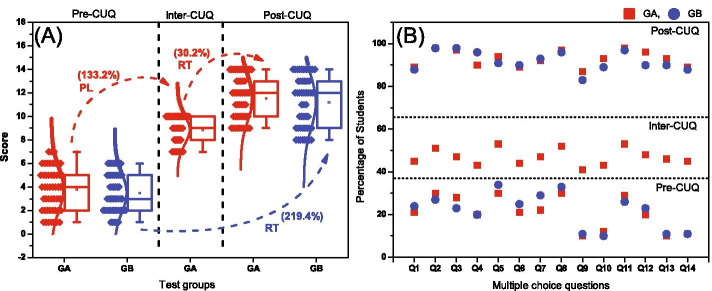
A Box plot showing the range of scores of 14 CUQ of Group A and Group B. Percentages in the brackets showing the percentage increase of average score in each tests. B Figure showing the percentage of students who got correct answers for each MCQs in each tests

GA students were assessed using the CUQ questionnaire after performing the experiment in PL (intermediate-CUQ) to understand the level of CU of students (called GA-inter). The results show that there is a significant difference (p < 0.05) when compared to the average score of GA-pre (CU score of GA before performing PL) as a result of physical lab experimentation. Similarly, the average score of GA-inter is significantly different from GB-pre (CU score of GB before performing RT-UTM).

The students were again assessed using the same questionnaire after performing the experiment in PL+RT (GA) and RT-UTM (GB) (Fig. 12). The results suggest a significant improvement in the conceptual understanding of both GA-post and GB-post students after performing RT-UTM experiments compared to the pre-experiment (GA-pre and GB-pre) data and the GA-inter data post-physical lab only, although no significant difference was found between the two post-experiment groups (GA-post and GB-post). This illustrates the benefit of incorporating RT-UTM intervention in Mechanics of Solids education to aid physical lab conceptual understanding (Corter et al., 2011; Lowe et al., 2013). Additionally, we compared the average CUQ scores of both groups before and after performing the experiment as shown in Fig. 13A. These box plots show the range of scores of students from minimum to maximum, the average score (solid horizontal line), four interquartile ranges, data points and distribution curves of both groups. Group A shows 133.2% increase in the average score after performing experiment in PL. A further increase of 30.2% in the average score of CUQ after performing the same experiment in RT platform is observed (A total of 203.7% increase in the average score from 3.80 to 11.54), confirming the t-test observation that RT-UTM intervention results in statistically significant improvement in conceptual understanding due to its self-paced personalized nature. Compared to GB-pre, GB-post shows 219.4% increase in the average score of CUQ after performing experiment in RT platform directly without physical lab, confirming the t-test observation that the benefit of RT-UTM intervention in physical lab education (RT + PL) is statistically indistinguishable from RT-only. This shows that RT + PL incorporates all the benefits of RT platform, like self-paced learning and personalized test visualization, which help in improving conceptual understanding.

**Fig. 14:**
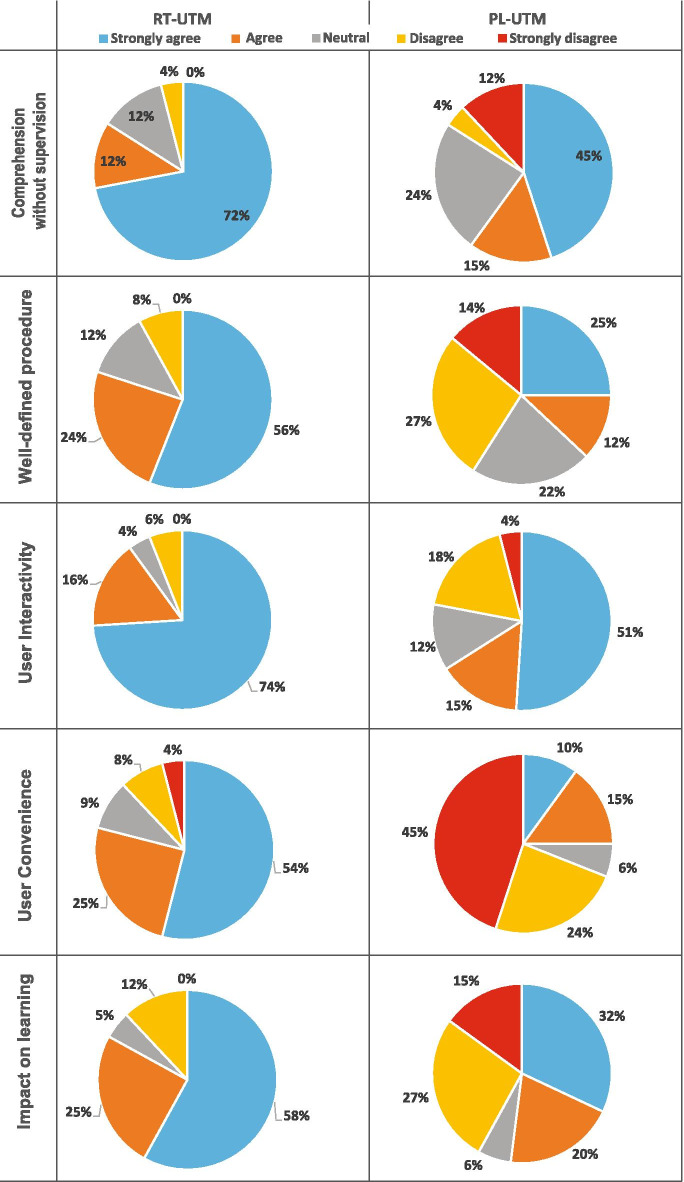
Effectiveness of RT-UTM and PL-UTM from the feedback data

The percentage of students who got correct answers for each MCQ is shown in Fig. 13B. Stress concentration due to the presence of notch on the specimen (Q9, Q10) and Poisson’s ratio (Q13, Q14) were the concepts students found most difficult to comprehend. These gaps in conceptual understanding were corrected after using RT-UTM platform due to more S-I and S-M interactions resulting in lower TD. Figure 13B shows only 40–60% of students were able to identify the correct answer for each question after the physical laboratory experimentation. But approximately 80%-90% of students were able to identify the correct answers for each question after the integration of RT-UTM into the study.

### Effectiveness of PL-UTM and RT-UTM platforms

The effectiveness of learning platform feedback assesses the following usage-related critical aspects of UTM experimentation: (1) comprehension without supervision, (2) a well-defined procedure (3) user interactivity, (4) user convenience and (5) impact on learning. Figure 14 shows the students’ feedback after performing PL-UTM (Group A) and RT-UTM (Group B). The responses from students indicate that majority of students either strongly agreed or agreed that RT-UTM is effective in all of above five aspects. From the feedback, 84% of the students agreed or strongly agreed that RT-UTM helped them comprehend the concepts and run the experiments without supervision and 60% of the participants reported that physical lab helped them comprehend the concepts and run the experiments without supervision. In the physical lab, students need to interact with the instructor to gain knowledge about the experiment. But in RT-UTM, contents like animated videos, simulated experimental setup, and video lectures add variety and individualization to help students understand the concepts without supervision. Students are able to review the RT-UTM learning material multiple times without time constraints until adequate comprehension is exhibited in self-assessment. This increases the flexibility of the structure and lowers the transactional distance. More than 80% of the students agreed that the RT-UTM had useful step-by-step procedure that aided in the execution of experiments. When comparing with RT-UTM, only 37% of the participants responded that physical lab has well-defined procedure. The rigid time-bound structure of physical lab does not allow self-paced learning prior to self-assessment of conceptual and procedural clarity. A significant number of users (i.e., over 90%) felt that RT-UTM had good user interactivity resulting in satisfactory user experience. The interactive graphical user interface of RT-UTM allows students to control the loading and unloading speed of the experimentation and allows real-time data visualization and capture. More than 79% of the students agreed that RT-UTM is convenient to perform the experiments. This can be explained by the higher functionality, media richness, and usability of RT-UTM platform. Impact of learning score shows the average of all the above factors, which represents how much the structure of RT/PL helps students to attain the goal of the experimentation. About 83% of the students either strongly agreed or agreed that RT-UTM had an impact on their learning outcomes where as, in physical lab, only 52% of the students strongly agreed or agreed that the structure of PL had an impact on their learning outcomes. RT-UTM allows students to perform and repeat the experiments at their convenience, enhancing flexibility in structure in addition to higher S-I and S-M interaction. This enhances autonomy and reduces the transactional distance, resulting in effective learning.

## Conclusion

A significant gap in physical laboratory education that stems from the lack of individualized learning has been addressed in this work through development of virtual laboratory (VL) platform that allows students to experiment individually and remotely through online access. A collaborative approach to laboratory education in Mechanical Engineering using both physical laboratory demonstrations and remote one-on-one laboratory sessions on the physical hardware can harness the benefits of both paradigms. This work successfully demonstrated the architecture and design enhancements required on one of the most critical experimental hardware in mechanics of solids laboratory i.e., Universal Testing Machine to enable a variety of remote experiments such as determination of Young’s modulus, estimation of Poisson’s ratio, stress concentrations experiments using hole and notch specimens and study of Saint Venant’s principle effectively by providing access and learning in a self-paced manner. The work also delves into the critical factors that impact remote laboratory learning such as student-instructor interactions as well as student-machine interaction. The multi-modal virtual laboratory RT-UTM platform was augmented with videos and quizzes and allowed users to perform interactive experimentation by controlling experimental parameters in real-time and visualizing the resulting data. Remote learners repeated experiments up to 3 times more and had more frequent interactions with instructors on questions related to theory and experimental procedures, along with higher student-machine interaction. The high accessibility and flexibility to re-do experiments along with interactive sessions with peers and instructors contributed to improved learning within remote laboratory environment.

To evaluate the effect of the platform for meeting the learning outcome, two survey instruments i.e., (1) Conceptual Understanding Questionnaire (CUQ) and (2) Effectiveness of VL Platform were developed as part of this study. Each survey instrument had specific assessment objectives. Through statistical analysis, CUQ effectively captured the conceptual understanding and meeting of learning objectives in both environments i.e. physical laboratory and RT-UTM. A significant improvement in the percentage of students who got correct answers (80–90%) for each multiple choice question was observed when physical laboratory was supplemented with RT-UTM virtual laboratory experimentation. The study also shows an overall improvement of 200% in the conceptual understanding of students after the integration of RT-UTM. In terms of Transactional Distance Theory, the higher conceptual understanding is effected by low transactional distance through lower structure, higher student-instructor and student-machine interaction, and higher autonomy.

Through the ‘Effectiveness of VL platform’ survey instrument, the extent of a user’s familiarity with experimental procedural details, experiences with the remote user interface and online content were captured. Encouraging feedback was also obtained from the ease of use study with 54–72% agreeing that the platform enormously helped them with comprehension and procedural thoroughness in comparison to those not exposed to RT-UTM platform. The higher flexibility (low structure) in terms of variety, individualization, media use, visualization, and usability contribute to increased effectiveness reported by the users through lowering of the transactional distance.

This work has significant implications for academic institutions and teachers in providing and enabling a learning environment that promotes effective individualized learning and comprehension of experimental concepts and skills impacting laboratory educational outcomes.

## Supplementary Information

**Additional file 1.** Conceptual Understanding Questionnaire.

**Additional file 2.** Effectiveness Survey Instrument.

## Data Availability

The data that support the findings of this study are available from the corresponding author upon reasonable request.
