# Supplementary material for: Impact of remote experimentation, interactivity and platform effectiveness on laboratory learning outcomes
Source: Int J Educ Technol High Educ. 2021 Jul 8;18(1):38. doi: 10.1186/s41239-021-00272-z (PMC8263093; doi:10.1186/s41239-021-00272-z)
Supplement: Supplementary file 1 — Additional file 1. Conceptual Understanding Questionnaire. [file 41239_2021_272_MOESM1_ESM.pdf]

Conceptual Understanding Questionnaire (CUQ)

Q1. The Young's modulus, in the linear stress-strain region, is...

a. dependent on load
b. dependent on strain
c. constant
d. variable

Q2. The experiment does not allow the specimen to yield because of...

a. strain hardening
b. failure at yield
c. machine incapable of yield load
d. gauge inaccurate at yield

Q3. Engineering stress differs from True stress because of...

a. Nominal Initial Area
b. Necking Area
c. Failure Area
d. Yield Area

Q4. The strain in the experiment is measured using strain gauge instrumentation because...

a. strain gauge has infinite life
b. gauge installation is very easy
c. micro-strain elongation
d. gauges cheaper than encoder

Q5. The point at which the linear stress-strain relationship ends is called...

a. ultimate strength
b. yield
c. proportional limit
d. 0.2% yield

Q6. The stress concentration due to hole is expected to be...

a. same as average stress
b. 10 times average stress
c. 2.5 times average stress
d. 100 times average stress

Q7. If the nominal stress for ductile material reaches the failure limit, the stress concentration factor is...

a. Eliminated
b. Doubled
c. Exponential
d. Halved

Q8. Stress Concentration around a Notch experiment is an example of...

a. Average loading
b. Mode 1 loading
c. Model 3 loading
d. Torsional loading

Q9. What is the notch stress to average stress ratio?

a. 1000
b. 100
c. 50
d. 2 – 4

Q10. The notch tip Stress intensity factor is a function of...

a. equal to crack length
b. square of crack length
c. log of crack length
d. square root of crack length

Q11. In Saint Venant's Principle a typical ratio of the concentrated stress to the nominal would be...

a. 1
b. 2.7
c. 4
d. 10

Q12. Saint Venant's Principle predicts near the load application point...

a. a higher stress than nominal
b. a lower stress than nominal
c. same stress as nominal
d. yield stress value

Q13. The negative lateral strain corresponds to...

a. compression in lateral direction
b. compression in longitudinal direction
c. stress concentration
d. yield phenomena

Q14. The shear modulus is a function of the Poisson's ratio and the...

a. Flexural Rigidity
b. Young's Modulus
c. Specimen length
d. Yield Stress
