# Supplementary material for: Impact of remote experimentation, interactivity and platform effectiveness on laboratory learning outcomes
Source: Int J Educ Technol High Educ. 2021 Jul 8;18(1):38. doi: 10.1186/s41239-021-00272-z (PMC8263093; doi:10.1186/s41239-021-00272-z)
Supplement: Supplementary file 2 — Additional file 2. Effectiveness Survey Instrument. [file 41239_2021_272_MOESM2_ESM.pdf]

Effectiveness Survey Instrument

RT-UTM	PL-UTM
Comprehension without supervision	
I know how to measure the strain in order to determine the Young’s modulus in RT UTM.	I know how to measure the strain in order to determine the Young’s modulus in PL UTM.
Using the zoomed-in animation, I am able to comprehend the specimen elongation	In PL-UTM, I am able to comprehend specimen elongation.
I know that the compression or tension of the same specimen can help me determine Young's modulus.	I know that the compression or tension of the same specimen can help me determine Young's modulus.
Well defined procedure	
Experiment procedure provided in the RT-UTM gives me a good idea about carrying out the experiment.	Experiment procedure provided in the PL-UTM gives me a good idea about carrying out the experiment.
Even though the specimen comes preloaded in the RT-UTM experimental setup, I am able to comprehend the loading process.	Even though the instructor loads the specimen and demonstrates the experiment in PL, I am able to comprehend the loading process.
I am able to get a proper understanding of the experimental calculations involved in the UTM experiments.	I am able to get a proper understanding of the experimental calculations involved in the UTM experiments.
User Interactivity	
I am able to interact with RT instrument much more than what I do in the physical lab (PL).	I am able to interact with UTM instruments in PL effectively.
RT-UTM allows me to take control over the instrument to a greater extent than I do in PL.	PL-UTM allows me to take control over the instrument to a greater extent.
Learning of experiments through RT-UTM was fun, engaging and effective	Learning of experiments through PL-UTM was fun, engaging and effective.
User Convenience	
I find RT-Virtual Lab (RT-UTM) easy to access.	I find PL-UTM easy to access.
The contents of RT-UTM are well organised and easy to navigate.	The contents of PL-UTM are well organized and easy to follow.

I can repeat the experiments at my convenience in RT-UTM.	I can repeat the experiments at my convenience in PL-UTM.
Impact on Learning	
I am able to get a proper understanding of the experiment in RT-UTM.	I am able to get a proper understanding of the experiment in PL-UTM.
Performing experiment in RT-UTM increases my confidence level for performing the same in PL	Performing experiments in PL-UTM increases my confidence level for unsupervised experimentation
RT-UTM has helped improve my performance in PL	PL-UTM has helped me to improve my performance in the laboratory.
